# Vector competence of *Aedes aegypti* and *Culex quinquefasciatus* from the metropolitan area of Guadalajara, Jalisco, Mexico for Zika virus

**DOI:** 10.1038/s41598-019-53117-1

**Published:** 2019-11-18

**Authors:** Darwin Elizondo-Quiroga, Miriam Ramírez-Medina, Abel Gutiérrez-Ortega, Armando Elizondo-Quiroga, José Esteban Muñoz-Medina, Gustavo Sánchez-Tejeda, Cassandra González-Acosta, Fabián Correa-Morales

**Affiliations:** 1 Unidad de Biotecnología Médica y Farmacéutica, Centro de Investigación y Asistencia en Tecnología y Diseño del Estado de Jalisco, Guadalajara, Jalisco Mexico; 2 Secretaria de Salud Jalisco, Guadalajara, Jalisco Mexico; 3 Laboratorio Central de Epidemiología, Centro Médico Nacional La Raza, Instituto Mexicano del Seguro Social, Mexico City, Mexico; 4 Dirección del Programa de Enfermedades Transmitidas por Vector, Centro Nacional de Programas Preventivos y Control de Enfermedades, Secretaría de Salud de México, Mexico City, Mexico

**Keywords:** Viral transmission, Viral vectors

## Abstract

Zika virus (ZIKV) is a mosquito-borne pathogen discovered in the late 40’s in Uganda during a surveillance program for yellow fever. By 2014 the virus reached Eastern Island in the Americas, and two years later, the virus spread to almost all countries and territories of the Americas. The mosquito *Aedes aegypti* has been identified as the main vector of the disease, and several researchers have also studied the vector competence of *Culex quinquefasciatus* in virus transmission. The aim of the present study was to evaluate the vector competence of *Ae*. *aegypti* and *Cx*. *quinquefasciatus* in order to understand their roles in the transmission of ZIKV in Guadalajara, Jalisco, Mexico. In blood feeding laboratry experiments, we found that *Ae*. *aegypti* mosquitoes showed to be a competent vector able to transmit ZIKV in this area. On the other hand, we found that F0 *Cx*. *quinquefasciatus* mosquitoes are refractory to ZIKV infection, dissemination and transmission.

## Introduction

Zika virus (ZIKV) was discovered in 1947 in Uganda, Africa during a surveillance program for yellow fever, where it was isolated from a sentinel Rhesus monkey in the Zika forest. By the following year, it was isolated from the *Aedes africanus* (Theobald) mosquito in the same region^1^. For more than 60 years the virus was circulating in Africa and Asia, but an outbreak in 2007 on Yap Island (Micronesia) marked the beginning of the spread of the virus out of those continents^2^. By 2014 the first report from a territory closer to the Americas was recorded on the Eastern Island located in the South Pacific, more than 2000 miles from the mainland of Chile, with a total of 173 Zika cases in that year^3^. By 2015, the first locally acquired Zika disease case in the mainland Americas was reported in Brazil^4^, but later evidence using negative samples to dengue infection from Rio de Janeiro, showed that ZIKV has been circulating since April 2013^5^.

After the appearance of ZIKV inland, it took just a couple of years to spread to almost all tropical and subtropical countries and territories of the continent^6^. Since the presence of the mosquito *Ae*. *aegypti* (L.) (a species considered to be the main ZIKV vector in urban and suburban areas)^7,8^ is well known in most of these areas, it was expected that this species would be the main vector responsible for the overwhelming spread of the virus. Evidence from large outbreaks in Brazil and Mexico in 2015 support this conclusion. In these outbreaks, it was found that natural infection with ZIKV was achieved in just *Ae*. *aegypti* mosquitoes^9,10^. It is, however, well known that more species are involved in virus transmission in many countries around the world. In fact, in some countries other mosquito species are considered to be the main vectors of the virus. These include mosquitoes from different genera, such as *Culex, Mansonia, Anopheles*, where some of these species are involved in the enzootic cycle and not in urban transmission^8^. Based on this, several researchers have studied the role of *Ae*. *aegypti* and other mosquitoes in the explosive outbreaks of ZIKV after its escape from Africa and Asia.

Assessing vector competence in *Ae*. *aegypti* mosquitoes for ZIKV transmission from the Americas has yielded diverse results (infection rates ranging 0–90%). For instance, Main, *et al*. found infection rates (IR) ranging from 81–90% in this species, using three different ZIKV strains and concluded that *Ae*. *aegypti* from California is an efficient vector for transmitting both ancestral and contemporary Asian lineages of the virus in laboratory studies^11^. On the other hand, Chouin-Carneiro *et al*. found high levels of infection but moderate to low dissemination and transmission levels in *Ae*. *aegypti* and *Ae*. *albopictus* (Skuse) from the Americas using the Asian lineage of the virus. They concluded that, although these mosquitoes were susceptible to the infection, they were low competent vectors for ZIKV^12^. In a different study, Zimler and Alto, tested *Ae*. *agypti* and *Ae*. *albopictus* from Florida, with three different doses of ZIKV, concluding that using higher doses of the virus resulted in overall greater infection rates for both species^13^. Weger-Lucarrelli *et al*. assessed the replication of three ZIKV strains in several cell lines in order to analyze the vector competence of *Ae*. *aegypti*, and they found that the three strains can replicate in *Ae*. *aegypti* mosquitoes from Mexico^14^.

Regarding *Culex quinquefasciatus* (Say) mosquitoes from the Americas, experimental studies have shown opposite or controversial results. Some have reported this species is not competent or even refractory to ZIKV infection. Experimental or field evaluations, however, have shown replication even at the salivary glands. Fernandes *et al*. demonstrated in two different studies that infection rates were minimal to completely absent in *Cx*. *quinquefasciatus* and dissemination and transmission were not detected^15^, concluding for both studies that this species is refractory to ZIKV^15,16^. Similar results regarding this species were reported by Main *et al*.^11^. On the other hand, Guedes *et al*. found that *Cx*. *quinquefasciatus* from Recife, Brazil can transmit the virus in laboratory conditions, and they were able to detect the virus in mosquitoes collected from different urban areas, where there was a high incidence of microcephaly^17^. However, Roundy *et al*. pointed out that viral RNA levels measured in these Recife pools had high RT-qPCR CT values (37.6–38.15), which does not correspond to transmission-competent mosquitoes^18^. In a different study, Smartt *et al*. reported that reared *Cx*. *quinquefasciatus* mosquitoes can be vectors of the virus, since they found ZIKV RNA in bodies and saliva from the assessed mosquitoes^19^. Weger-Lucarelli *et al*. found the opposite in laboratory colonies of *Cx*. *quinquefasciatus*, where they observed very little to no infection with ZIKV^14^.

In a previous study, we reported ZIKV presence in mosquito salivary glands and other body parts of five wild-caught female species, including *Ae*. *aegypti* and *Cx*. *quinquefasciatus*, and also in male mosquitoes for these two species in the metropolitan area of Guadalajara, Jalisco, Mexico^20^. *Ae*. *aegypti* and *Cx*. *quinquefasciatus* are the main mosquito species present in urban and suburban areas in the state of Jalisco. Since we found the virus in these two species in the wild, the aim of the present study is to evaluate them for vector competence in laboratory conditions in order to understand their roles in the transmission of the ZIKV.

## Results

### Mosquito oral infections

A total of 492 female mosquitoes were fed on eleven blood meal dates, and 412 survived for dissection and saliva collection at 14 dpi (some dissections were performed at 13 dpi and, in one case, 10 dpi) (Table 1). From these 412 mosquitoes, 270 (65.5%) were *Ae*. *aegypti* and 142 (34.4%) *Cx*. *quinquefasciatus*. Blood meals provided to the mosquitoes were performed using ZIKV strains with different virus titers expressed as TCID_50_/mL, in three different orders of magnitude (10^4^, 10^5^, and 10^6^) designated as low, medium and high virus concentrations (Table 1). From all 412 mosquitoes, saliva was collected, and each mosquito was dissected to measure transmission, infection and dissemination of the virus; mosquito body parts were ground with plastic pestles. All samples were mixed in the medium by pipetting, and supernatants were inoculated into individual wells from a 48 well plate containing confluent Vero cells cultures, looking for CPE caused by the presence of the virus in the mosquito homogenates. Of these, 289 from *Ae*. *aegypti* and one from *Cx*. *quinquefasciatus* inoculations showed CPE. More specifically, at medium virus concentration, a total of 82 *Ae*. *aegypti* mosquitoes showed CPE, while at the higher virus concentration 207 *Ae*. *aegypti* and 1 *Cx*. *quinquefasciatus* showed CPE. No CPE was observed in the lower virus concentration for both species (Table 2).

**Table 1:** Fed mosquito batches for both species and viral concentration used in the experiment.

Batch	Viral titer in TCID_50_/mL	Fed Mosquitoes	Days post infection (dpi)	Processed Mosquitoes
1	TCID_50_/mL = 1.58E^+5^	9 *Ae*.	10	2 *Ae*.
	TCID_50_/mL = 4.64E^+4^	27 *Cx*.	14	22 *Cx*.
2	TCID_50_/mL = 1.58E^+5^	29 *Ae*.	13	12 *Ae*.
3	TCID_50_/mL = 1.58E^+5^	41 *Cx*.	14	37 *Cx*.
4	TCID_50_/mL = 3.73E^+5^	20 *Ae*.	14	15 *Ae*.
	TCID_50_/mL = 4.64E^+4^	24 *Ae*.	14	24 *Ae*.
5	TCID_50_/mL = 3.73E^+5^	28 *Ae*.	14	22 *Ae*.
6	TCID_50_/mL = 4.64E^+4^	13 *Ae*.	13	13 *Ae*.
	TCID_50_/mL = 2.68E^+5^	28 *Ae*.	13	18 *Ae*.
7	TCID_50_/mL = 1.58E^+5^	17 *Ae*. 39 *Ae*.	14	17 *Ae*. 31 *Ae*.
8	TCID_50_/mL = 2.68E^+6^	31 *Ae*. 5 *Cx*.	14	30 *Ae*. 4 *Cx*.
9	TCID_50_/mL = 2.68E^+6^	21 *Ae*. 40 *Cx*.	14	21 *Ae*. 33 *Cx*.
10	TCID_50_/mL = 2.68E^+6^	47 *Ae*. 18 *Cx*.	14	46 *Ae*. 14 *Cx*.
11	TCID_50_/mL = 2.68E^+6^	19 *Ae*. 36 *Cx*.	13	19 *Ae*. 32 *Cx*.
Total:		**325 Ae**. **167 Cx** **Total: 492**		**270 Ae**. **142 Cx**. **Total: 412**

**Table 2:** Total of mosquito homogenates in the different virus concentrations, showing CPE and infection, dissemination and transmission rates for both species.

Virus Conc.	Species	Processed mosquitoes	B	H	S	% IR	% DR	% DIR	% TR	% TE
Low 10^4^	*Ae*. *aegypti*	37	0	0	0	0	0	0	0	0
	*Cx*. *quinquefasciatus*	22	0	0	0	0	0	0	0	0
Medium 10^5^	*Ae*. *aegypti*	117	45	28	9	38.46	23.93	62.22	32.14	7.69
	*Cx*. *quinquefasciatus*	37	0	0	0	0	0	0	0	0
High 10^6^	*Ae*. *aegypti*	116	108	83	16	93.10	71.55	76.85	19.28	13.79
	*Cx*. *quinquefasciatus*	83	0	0	1	0	0	0	0	1.20
Total			***Ae***. **153** ***Cx***. **0**	***Ae***. **111** ***Cx***. **0**	***Ae***. **25** ***Cx***. **1**					

Infection rate (IR) = infected mosquito bodies/number of mosquitoes tested.

Dissemination rate (DR) = infected mosquito heads/number of mosquitoes tested.

Disseminated infection rate (DIR) = infected mosquito heads/infected mosquito bodies.

Transmission rate (TR) = mosquitoes with infected saliva/infected mosquito heads

Transmission efficiency (TE) = mosquitoes with infected saliva/number of mosquitoes tested.

All rates are multiplied by 100 to obtain percentages.

### Susceptibility to ZIKV infection

**Figure 1:**
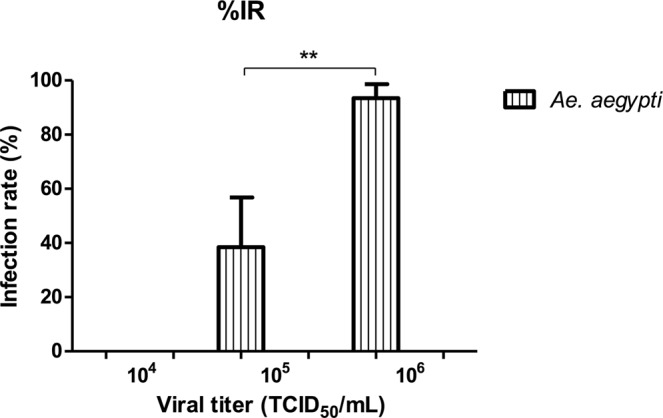
Susceptibility to ZIKV infection was expressed as infection rates (IR = infected mosquito bodies/number of mosquitoes tested) for each virus concentration. The data are presented as mean ± SD. Asterisks show the significant difference between the rates obtained with medium and high virus concentrations for *Ae*. *aegypti* (**p < 0.01). For *Cx*. *quinquefasciatus* no infection was found in any of the viral titers. Statistical analysis was performed using Student’s t-test.

The mosquito body homogenates were assessed for ZIKV infection in cell culture looking for CPE. The infection rate (IR) was calculated with the number of infected mosquito bodies divided by the total tested. In the lowest virus concentration, 37 bodies corresponding to *Ae*. *aegypti* and 22 to *Cx*. *quinquefasciatus* were assessed. None presented CPE, resulting in zero infection rates. In the medium virus concentration, out of 117 engorged *Ae*. *aegypti*, 45 bodies showed CPE resulting in an infection rate of 38.46%. For *Cx*. *quinquefasciatus* 37 bodies were evaluated and none produced CPE (0%). For the highest virus concentration, *Ae*. *aegypti* showed an infection rate of 93.10% (108 bodies), and for *Cx quinquefasciatus* 83 mosquitoes were evaluated without the appearance of CPE (zero infection rates) (Table 2). Statistical analysis showed significant differences (p < 0.01) between the rates obtained with medium and high virus concentrations for *Ae*. *aegypti* mosquitoes (Fig. 1). For *Cx*. *quinquefasciatus* there was no infection, and therefore no differences were found in any of the viral titers. The calculation of minimum infection rate (MIR) was performed using the PooledInfRate v.4.0 program. Since this calculation is for mosquito pools ([number of positive pools/total specimens tested] x 1000) we use the mosquitoes from each feeding date as if they were mosquito pools; therefore, a total of 11 pools were formed and separated according to the viral titer used (10^4^, 10^5^, 10^6^) to calculate MIR. For *Ae*. *aegypti* mosquitoes, MIR of 42.74 and 34.48 were found for the medium and high virus concentration, respectively (Table 3).

**Table 3:** Minimum Infection Rate values for Aedes aegypti; in the case of Culex. quinquefasciatus all values were null.

Viral titer	MIR/1000	MIR %	Lower limit	Upper limit
***Ae. aegypti***				
10^4^	0.00	0.00	0.00	54.88
10^5^	42.74	4.274	6.09	79.38
10^6^	34.48	3.448	1.28	67.69
General	33.33	3.333	11.92	54.74

MIR = (number of positive groups/total of analyzed mosquitoes) × 1000.

### Dissemination of ZIKV infection in mosquitoes

**Figure 2:**
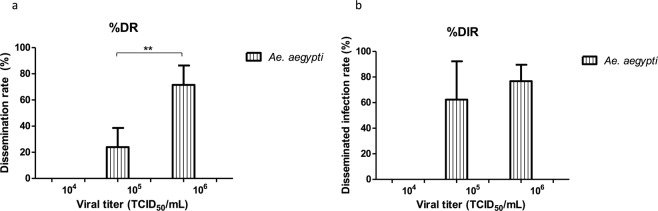
The dissemination rate (DR = infected mosquito heads/number of mosquitoes tested) and the disseminated infection rate (DIR = infected mosquito heads/infected mosquito bodies) were calculated. The data are presented as mean ± SD. (**a**) Asterisks show the significant difference between dissemination rates obtained with medium and high virus concentrations for *Ae*. *aegypti* (**p < 0.01). (**b**) In DIR, no significant differences were found for *Ae*. *aegypti*. In the case of mosquitoes *Cx*. *quinquefasciatus* DR and DIR do not differ significantly. Statistical analysis was performed using Student’s t-test.

For dissemination, the mosquito head homogenates were analyzed in cell culture looking for CPE. The dissemination rate (DR) was calculated with the number of infected mosquito heads divided by the total tested, and the disseminated infection rate (DIR) was calculated with the number of infected mosquito heads divided by the infected mosquito bodies. In the lowest virus concentration, no dissemination was observed for both species. In the medium virus concentration, 28 heads of *Ae*. *aegypti* showed CPE resulting in 23.93% of DR and 62.22% of DIR. Again, no presence of CPE was observed for *Cx*. *quinquefasciatus* mosquitoes. For the high virus concentration in the case of *Ae*. *aegypti*, 71.55% of DR and 76.85% of DIR were obtained (83 positive heads). In *Cx*. *quinquefasciatus* the dissemination rates were once again 0% (Table 2). For *Ae*. *aegypti*, when statistically comparing the ranges of DR obtained in medium and high virus concentrations (10^5^ and 10^6^), significant differences were observed (p < 0.01) (Fig. 2a). On the other hand, no significant differences were observed in DIR between the titers of 10^5^ and 10^6^ (Fig. 2b). In the case of *Cx*. *quinquefasciatus* mosquitoes, no significant differences were observed for DR and DIR since both parameters were 0% (Fig. 2a,b).

### ZIKV transmission

**Figure 3:**
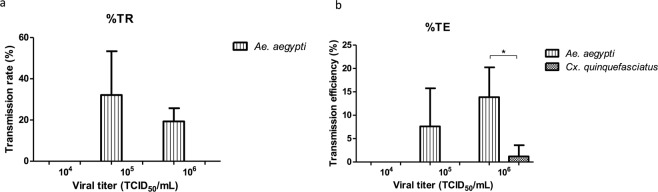
The transmission rate (TR = mosquitoes with infected saliva/infected mosquito heads) and transmission efficiency (TE = mosquitoes with infected saliva/number of mosquitoes tested) were calculated. The data are presented as mean ± SD. (**a**) In TR, no significant differences were found for *Ae*. *aegypti* (Student’s t-test). (**b**) In TE, the asterisk shows the significant difference between *Ae*. *aegypti* and *Cx*. *quinquefasciatus* with the highest viral load (*p < 0.05, one-way ANOVA and Tukey’s HSD *post hoc* tests).

Mosquito saliva was analyzed in cell culture looking for CPE in order to better understand the transmission of ZIKV. Estimation of transmission rate (TR) was calculated using the number of mosquitoes with infected saliva divided by infected mosquito heads, and transmission efficiency (TE) was calculated using the number of mosquitoes with infected saliva divided by the number of mosquitoes tested. In the lowest virus concentration, no transmission was observed for both species. In the medium virus concentration for *Ae*. *aegypti*, 9 positive saliva were found (TR of 32.14% and a TE of 7.69%) and for *Cx*. *quinquefasciatus* both rates were zero. For the high virus concentration in the case of *Ae*. *aegypti*, 16 saliva showed CPE (19.28% of TR and 13.79% of TE); in *Cx*. *quinquefasciatus* a single saliva showed CPE (TR of 0% and TE of 1.20%) (Table 2). In the case of *Ae*. *aegypti*, statistical analysis showed no significant differences for both TR and TE in medium and high virus concentrations (10^5^ and 10^6^)(Fig. 3a,b). On the other hand statistical differences were found (p < 0.05) when comparing the TE values between *Ae*. *aegypti* and *Cx*. *quinquefasciatus*, in the high virus concentration (10^6^) (Fig. 3b).

### Viral load in Saliva

**Figure 4:**
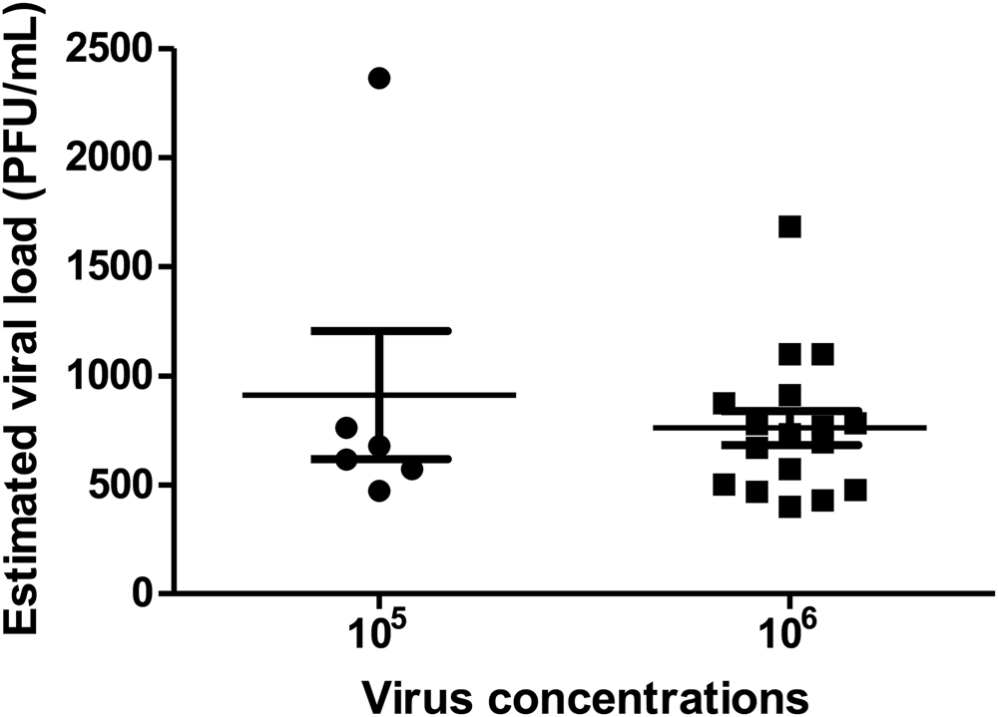
ZIKV estimated titers in saliva expressed in PFU/ml from mosquitoes fed with two different virus concentrations (10^5^ and 10^6^). Saliva titers were calculated using CT mean from RT-qPCRs interpolating data into a standard curve from a ZIKV sample with a known titer. The saliva data are presented as mean ± SEM.

In order to estimate the ZIKV load and titer expectorated in mosquito saliva, RT-qPCRs were performed using RNA extractions directly from the original saliva tube. Standard curves were designed using RNA extraction from a ZIKV sample with a viral titer of (2.68 × 10^6^ TCID_50_/mL) to compare titers in saliva. All PCR reaction were performed in triplicate. Out of the 26 saliva that yielded CPE in cell cultures, 23 nucleic acids extracted from the original tube were detected by the RT-qPCR reactions, including the single saliva that yielded CPE in *Cx*. *quinquefasciatus*. Using CT results of the saliva, the viral titer in both TCID_50_/mL and the estimated PFU/mL per sample were calculated. The viral loads were in a range between 569-2403 TCID_50_/mL_,_ corresponding to 398-1682 PFU/mL (Table 4). Out of the 23 detected silivas by RT-qPCR, six were detected in the medium virus concentration for *Ae*. *aegypti* and 17 were detected in the high virus concentration, corresponding 16 for *Ae*. *aegypti* and one for *Cx*. *quinquefasciatus* (Fig. 4).

**Table 4:** Ct mean values (RT-qPCR by triplicate) and estimated saliva viral titer expressed in in TCID_50_/mL and PFU/mL.

Batch	Sample	Mean Ct.	Standard deviation (SD)	TCID_50_/mL	PFU/mL
5 (***Ae. aegypti***)	S 15	22.10	0.2254	880	616
	S 16	20.45	0.4801	3377	2364
	S 18	21.98	0.2707	970	679
	S 22	21.84	0.0300	1088	762
6 (***Ae. aegypti***)	S 2	22.19	0.2701	815	571
7 (***Ae. aegypti***)	S 22	22.43	0.4970	674	472
8 (***Ae. aegypti*** and ***Cx quinquefasciatus***)	S 11	21.95	0.0321	992	694
	S 15	22.63	0.0252	569	398
	S 16	22.55	0.1650	611	428
	S 20	21.89	0.0153	1047	733
	S 21	22.41	0.2991	681	477
	S 25	22.19	0.1929	817	572
	S 26	21.81	0.2237	1118	783
	S 4 *Cx*.	22.35	0.3365	717	502
9 (***Ae. aegypti***)	S 1	22.00	0.1856	957	670
	S 4	22.44	0.2427	666	467
	S 18	21.39	0.5979	1567	1097
	S 20	20.87	0.0700	2403	1682
10 (***Ae. aegypti***)	S 27	21.39	0.5807	1567	1097
	S 39	21.62	0.1682	1302	912
	S 46	21.83	0.1253	1097	768
11 (***Ae. aegypti***)	S 1	21.67	0.1963	1247	873
	S 16	21.81	0.1914	1112	778
Standard curve	Undiluted	12.24	0.2722	2.68 × 10^6^	1.88 × 10^6^
	Dilution 10^−1^	14.84	0.1311	268000	187600
	Dilution 10^−2^	18.55	0.4661	26800	18760
	Dilution 10^−3^	20.40	0.1193	2680	1876

0.7 PFU = 1 TCID_50_.

## Discussion

The viral loads for ZIKV on infected hosts varies through the time of infection^21^. Depending on viral titer in a bloodmeal, mosquito vectors will acquire different loads of virus that may or may not lead to infection^22^. ZIKV infected patients, usually present low viremia levels ranging from 10^3^ to 10^6^ RNA copies/mL^23^; because of this, the viral titers used in this study were of three different orders of magnitude (10^4^, 10^5^, and 10^6^) starting from the first viral load that is 100-fold higher than viremia reported for patients, trying to assure mosquito infections, and we designated them as low, medium and high virus concentrations in feeding experiments for both *Ae*. *aegypti* and *Cx*. *quinquefasciatus* mosquitoes; these virus concentrations were similar to those used in other studies. For instance, Fernandes *et al*. evaluated *Cx*. *quinquefasciatus* mosquitoes using a titer of 10^6^ PFU/mL^15^; Huang *et al*. also infected *Cx*. *quinquefasciatus*, but they used a higher order of magnitude, 10^7^ TCID_50_/mL^24^. Chouin-Carneiro *et al*. used the same viral order of magnitude (10^7^ TCID_50_/mL) and in this study they infected *Ae*. *aegypti* mosquitoes^12^. In another study carried out in Singapore, they analyzed both *Cx*. *quinquefasciatus* and *Ae*. *aegypti* mosquitoes, feeding them with 10^5^ and 10^6^ PFU/mL, and both species were infected. They then tested titers of different orders of magnitude in *Ae*. *aegypti*, (10^5^, 10^4^, 10^3^ and 10^2^ PFU/mL), and they proved that a viral load of 10^3^ PFU/mL was enough to infect the mosquito strains that they used for the experiments^25^. In our study, using a concentration of 10^4^ TCID_50_/mL (low virus concentration) to engorge both mosquito species, resulted in no infection, dissemination or transmission. When we used the medium virus concentration (10^5^ TCID_50_/mL), moderate to low rates of infection, dissemination and transmission were obtained for *Ae*. *aegypti*, where the highest rate was in the DIR, (dissemination in the mosquito bodies). For the high virus concertation (10^6^ TCID_50_/mL), a change was observed in all the rates, where both, infection and dissemination rates were high, and transmission rates low. But only IR and DR presented statistical differences. In general, it could be observed that exposing mosquitoes to a higher virus concentration resulted in overall greater infection rates for *Ae*. *aegypti*. This conclusion was previously observed in a study using this mosquito species from Florida in a dose-response experiment^13^. For all the different virus concentrations used in the present study for *Cx quinquefasciatus* mosquitoes, there was no infection or dissemination of the virus; only a single saliva was able to generate CPE in cell culture. Thus, it can be concluded that this species is refractory to ZIKV infection in our laboratory assays, as many other studies have concluded^11,14,15,26^.

In our study, MIR calculation was used for *Ae*. *aegypti* mosquitoes, and the obtained rates from medium and high virus concentration were higher (42.74 and 34.48, respectively) than those presented in a previous publication of our research group from wild caught *Ae*. *aegypti* mosquitoes where the MIR was 10.28, but were similar to other species evaluated in the same study^20^. In *Cx*. *quinquefasciatus* mosquitoes, all calculated MIRs were null.

Although *Ae*. *aegypti* is considered the main vector of the virus, previous studies of vector competence have produced contrasting results. Some have showed low to moderate percentages of virus competence^11,12,14,27–29^, but others have shown that this species is highly competent^14,15,25,30,31^. The results in our study are more similar to those in which ranges of infection and dissemination are high to moderate, but transmission rates are low.

The estimated viral titers that we found in the saliva of *Ae*. *aegypti* and from a single saliva sample in *Cx*. *quinquefasciatus* were predominantly in a range of 500–1600 PFU_/_mL, and these titers are similar to those described by Dudley *et al*., where they found a ZIKV dose delivered by a mosquito to be 10^1.5^ to 10^3.2^ PFU per mosquito^32^. Some researchers have reported that *in vitro* mosquito salivation overestimates the amount of virus inoculated, compared to virus deposited at sites of *in vivo* blood feeding for other arboviruses^33^, while other researchers have reported that mosquitoes inoculate high doses of WNV as they probe and feed on peripheral tissues in animal models^34^. So, to determine precisely the amount of ZIKV transmitted by mosquitoes, more studies are required, but in general it can be considered that titers found in our study could be enough to transmit the virus during a blood feeding.

Vector competence for ZIKV varies greatly. As has been published in our research, we found susceptibility to virus infection can be due to mosquito-virus match factors (also known as genotype-genotype interactions), including the source of blood used in experiments and viral load, among others. The role of immunity against the virus is not entirely clear. Although some mosquitoes are not considered highly competent vectors, they may be able to transmit the virus efficiently in the wild if they have an anthropophilic behavior.

In the case of saliva that were positive without presenting CPE in the body and head of the mosquitoes (one of *Cx*. *quinquefasciatus* and another of *Ae*. *aegypti*), this could be due to several factors involved to mosquito immunity, with a clarification phenomenon previously described. Some studies have described results in which the virus is not detectable in the midgut or the viral titers start to decrease, but the salivary glands of the same mosquito are positive^17,30^. In our study, ZIKV was found only in saliva but not in the head or body in one single pool from each species and we think this could be due to this phenomenon, nevertheless, another possibility could be related to a false positive result due to the high sensitivity of the assay. The clearance phenomenon has been described in both *Cx*. *quinquefasciatus* and in *Ae*. *agypti*, with ZIKV, DENV and WNV^35–38^. In the work of Liu *et al*., *Cx*. *quinquefasciatus* mosquitoes were found with infected midguts, where the viral titer was decreasing, and after 7 dpi, the virus was no longer detected. Although the clearance is not specifically mentioned, there is a possibility that it may be involved; the authors suggested that this phenomenon could have happened due to undigested infected blood. However, as little is known of vector competence of *Cx*. *quinquefasciatus* with ZIKV and the immune responses involved, these or other hypotheses may be involved here.

On the other hand, another hypothesis is that the blood source could also be involved in the results presented herein regarding the *Cx*. *quinquefasciatus* refractory tendencies to ZIKV infection. If mosquitoes had been fed with blood obtained directly from infected patients, the results could be different. It could be interesting to study this hypothesis, since in a previous publication from our group, ZIKV was isolated from salivary glands of wild *Cx*. *quinquefasciatus* mosquitoes and also from male mosquitoes from this species. In animal models for instance, Dudley *et al*. reported *Ae*. *aegypti* mosquitos acquiring ZIKV infection from a macaque infected by mosquito bites but not from macaques infected with a virus administrated by syringe, concluding that virus with different passage histories could result in genotypic and phenotypic differences^32^. Therefore, differences in the viral stocks we used for oral infection experiments could favor the infection in one mosquito species but not the other. A similar case using an animal model is mentioned in the article by Roundy *et al*., where they compared natural and artificial blood feeds. They observed that murine blood had greater infectivity than blood that was given in artificial feeds. They concluded that this difference in competence after blood exposure undoubtedly contributed to the underestimation of *Ae*. *aegypti* mosquitoes as a vector of ZIKV in previous studies^18^. Therefore, more studies are needed to understand the role of the cell lines used for virus amplification in the vector competence studies depending on the mosquito species.

In addition, factors such as environment, mosquito density, feeding patterns, mosquito survival rates (known as vector capacity), and others also influence the transmission of the virus. For example, Tabachnick *et al*. and Smartt *et al*. mention that there are environmental and biological conditions where *Cx*. *quinquefasciatus* mosquitoes are probably competent for ZIKV, and these mosquitoes may play a role in the transmission of the virus to humans^37,39^. Richard *et al*. showed that two *Aedes* species from French Polynesia did not show sufficient competence to transmit ZIKV and suggested that another mosquito species could contribute to the spread of the virus in that region. They also mentioned that *Cx*. *quinquefasciatus* is an abundant species that could have a role in the transmission of the virus^40^.

In our study, increasing the viral titers for the oral infections in *Ae*. *aegypti* mosquitoes showed increasing numbers in all rates. Although transmission rates were not significantly different, the increase of mosquitoes with ZIKV in the saliva could be observed, suggesting that this phenomenon could happen in the wild.

Even though *Cx*. *quinquefasciatus* mosquitoes can be found infected in nature (despite that reported viral titers were not according to those for transmitting the virus to humans), laboratory studies do not always manage to reproduce wild conditions, as happened in our study. However, there are publications where infection has been achieved, but the factors that influence the susceptibility in *Cx*. *quinquefasciatus* are not clear. Therefore, several analyses are needed in order to elucidate the exact role of this species in the cycle of transmission of the virus.

In conclusion, even when we detected the virus in salivary glands of wild *Cx*. *quinquefasciatus* mosquitoes in a previous study^20^, surprisingly, in the present work using F0 mosquitoes for oral blood feeding laboratory experiments, we found this species refractory to ZIKV infection, dissemination and transmission. Further analysis trying to explain this contrast should therefore be performed. On the other hand, in the case of *Ae*. *aegypti* mosquitoes, this species showed to be a competent vector to transmit ZIKV in the Guadalajara Jalisco metropolitan area.

## Methods

### Biosafety and ethical approval

This study was approved by the Centro de investigación y Asistencia en Tecnología y Diseño del Estado de Jalisco A.C. Biosecurity Committee (Third Ordinary session November 2016) and ethical approval by the National Scientific Research Committee from Instituto Mexicano del Seguro social (number R-2018-785012).

### Cell cultures and virus strains

For virus propagation and isolation, C6/36 mosquito cell line (ATCC^®^ CRL-1660^™^) and African green monkey kidney cells (Vero ATCC^®^ CCL-81^™^), both from American Type Culture Collection (ATCC^®^) were used in the study. Cells were propagated in L-15 medium (Leibovitz medium) at 28 °C, or DMEM (Dulbecco’s Modified Eagle’s medium) at 37 °C, for C6/36 or Vero cells, respectively, supplemented with 1% heat-inactivated fetal bovine serum (FBS), 1% penicillin/streptomycin, 1% glutamine, plus 1% non-essential amino acids in the case of C6/36 cells.

Three different ZIKV strains were used in this study for the oral exposure experiments, or as positive control on RT-qPCR reactions; two of them were isolated from wild caught *Ae*. *aegypti* and *Cx*. *quinquefasciatus* mosquitoes from the Guadalajara, Jalisco, metropolitan area in 2016, and these isolates were designated as *Ae*. G6, and *Cx*. The third strain used was isolated in CIATEJ from a serum sample previously confirmed as ZIKV by RT-qPCR at the Central Laboratory of Epidemiology, Mexican Institute of Social Security. The serum was inoculated into a 25 cm^2^ flask containing a confluent monolayer of C6/36 cells, and adsorbed for 1 hr. at 28 °C, rocking the flask every 15 min to distribute the inoculum; then L-15 medium containing 2% FBS was added, and incubated at 28 °C; the culture was observed under an inverted microscope daily until cytopathic effect (CPE) appeared. In order to confirm virus isolation, supernatant from the culture that showed CPE was filtered with 0.22um membrane disk and re-inoculated in fresh cells to confirm virus isolation by re appearance of CPE. The identity of all virus strains used in the study was confirmed at CIATEJ by RT-qPCR using the primers and probes previously reported by Lanciotti *et al*.^23^.

### Virus titration

In order to quantify the virus for the experiments, the tissue culture infectious dose 50 (TCID_50_) was calculated. Vero cells were seeded into 96-well plate and incubated overnight for adherence. 10-fold serial dilutions of viral supernatant from the second passage were prepared in L-15 medium containing 2% FBS. Then, 100 µL of these dilutions was inoculated per well using 8 replicates per dilution. After 5 days, the plate was analyzed looking for CPE. The viral titer was quantified as described by Reed and Muench^41^.

### Mosquitoes

*Ae*. *aegypti* and *Cx*. *quinquefasciatus* mosquito species were used for the vector competence experiments using the adults (F0) direclty from eggs or larvae collected from areas where no human confirmed cases were recored by health autorities, trying to reduce the posibility to emerged vertical ZIKV infected mosquitoes which could bias the results. Emerged mosquitoes were mantained in the CIATEJ insectary with the following conditions: 28 ± 1 °C with a light cycle: darkness of 12 h: 12 h and a relative humidity of 70%, approximately, until mosquito oral experimental infections. In the case of *Ae*. *aegypti*, eggs, larvae and adults of this species were provided by the Entomological Research Unit of the Department of Public Health of the State of Jalisco; for *Cx*. *quinquefasciatus*, larvae collections were carried out with personnel of this same department along with CIATEJ personnel.

### Mosquito oral infections

In order to determine vector competence, different mosquito batches were fed with human blood containing virus. Five to seven day old mosquito females were deposited in feeding boxes; 50 to 80 females extracted from maintenance cages of mosquito colonies by mechanical aspiration were placed in each box. Mosquitoes were kept without food for 48 hours, with a source of water (swabs with water) which was removed from the feeding boxes 24 hours before oral infections in order to ensure that a large proportion of females were fed. The food source in the feeding experiments was a mixture containing 1 mL of blood and 1 mL of viral suspension with a known viral titer, supplemented with adenosine triphosphate (ATP) as a phagostimulant, in a final concentration of 10 mM. The blood was obtained by venous puncture in vacutainer tubes with heparin as anticoagulant from volunteers involved in the project at CIATEJ. Infectious blood meals were performed using glass feeders connected to the water recirculation system at 37 °C, covered with a parafilm membrane (plastic film). Exposure time to bloodmeals was limited from 60 to 90 min. Engorged females were transferred to new feeding boxes and kept with swabs with 10% sucrose and water in the insectarium at 28 ± 1 °C, a light cycle: darkness of 12 h: 12 h and 70% humidity.

### Infection, dissemination and transmission analysis

In order to analyze infection, dissemination and transmission of ZIKV, batches of 10 to 60 mosquitoes were analyzed 14 days after infection (dpi). Only in some cases, when many mosquitoes died before reaching 14 dpi, was the dissection performed a few days earlier (most of the time 13 dpi and in one case 10 dpi) on the still alive mosquitoes. The number of mosquitoes per group depended on how many mosquitoes had fed in that batch. For each individual female mosquito, 3 different samples were collected: the body (B) (abdomen and thorax) to estimate the infection, the head (H) for virus dissemination and saliva (S), for virus transmission. Each of these samples was deposited into individual 1.5 tubes containing viral maintenance medium (200 μL of D-MEM medium supplemented with 20% FBS and 1% penicillin -streptomycin - amphotericin cocktail).

To obtain the three different samples per mosquito, subsets of mosquitoes per batch were knocked down by cold shock (−20 °C) for approximately 40–50 sec. or until they were fully anesthetized. Then, to collect saliva, wings and legs were removed and discarded immediately after the cold shock and the mosquito proboscis was inserted into a 30 μL microcapilar tube (Microcaps, Drummond Scientific Company, Broomall, PA, USA) or 25 μL microcapilar (Kimble® microcapillary pipettes, Sigma-Aldrich, St. Louis, MO, USA), containing viral maintenance medium. After 45 minutes, microcapillary tubes were placed into a tube with the medium. Subsequently, heads were separated from the body and each of these parts was placed into a different tube containing the same medium. Next, all tubes were centrifuged at 14,000 rpm for 1 min. and stored at −80 °C for further analysis. In order to identify the infection in the different samples, the tubes were thawed, the mosquito parts were ground in the same medium in which they were stored, and the resultant homogenates were centrifuged at 10,000 rpm for 10 min, including tubes with saliva. All centrifugations were performed in an eppendorf microcentrifuge 5417R (Eppendorf, Hamburg, Germany). Next, 50 μL of each supernatant (B, H, S) were inoculated into individual wells of a 48 well plate with Vero cells and incubated for 1 h at 37 °C. After this time, cell maintenance medium was added. The plates were incubated at 37 °C and examined daily for evidence of viral CPE for a maximum period of 21 days. The remaining homogenates were stored at −80 °C for further analysis.

### Viral analysis in positive samples

Once mosquito homogenate parts showed CPE in the cell culture, they were considered as positive to the virus; next, nucleic acid extraction was carried out directly from each homogenate stored at −80, using a QiAmp Viral RNA Mini Kit (Qiagen^™^, Hilden, Germany). In order to determinate presence of the virus and viral load in the original homogenates, RT-qPCRs were carried out in a Light Cycler 480 II PCR instrument (Roche Diagnostics, Penzberg, Germany) using the next 2 kits: Aedex^³^ Kit (Genes2life, Irapuato, Mexico) following the manufacturer’s protocol, and the primers and probe for viral load quantification included in the kit, and Verso 1-step RT-qPCR Kit (Termo FisherTM, MA, USA) using the primer pair and probe previously reported by Lanciotti *et al*.

### Statistical analysis

Minimum infection rates (MIR) were estimated using the PooledInfRate v.4.0 program. Likewise, infection, dissemination and transmission rates were calculated. Once the rates were obtained, comparisons were made to analyze the vectorial competence of the two species. All statistical analyses were conducted using the GraphPad prism software (version 5.0, La Jolla, CA, USA). Data were analyzed using the unpaired Student’s t-test, Analysis of variance (one-way ANOVA) and Tukey’s HSD *post hoc* tests. *p*-values ≤ 0.05 were considered statistically significant.
